# Comparing the Occurrence of Healthcare-Associated Infections in Patients with and without COVID-19 Hospitalized during the Pandemic: A 16-Month Retrospective Cohort Study in a Hospital Intensive Care Unit

**DOI:** 10.3390/jcm11051446

**Published:** 2022-03-07

**Authors:** Claudia Isonne, Valentina Baccolini, Giuseppe Migliara, Mariateresa Ceparano, Francesco Alessandri, Giancarlo Ceccarelli, Guglielmo Tellan, Francesco Pugliese, Maria De Giusti, Corrado De Vito, Carolina Marzuillo, Paolo Villari, Lavinia Camilla Barone, Dara Giannini, Daniela Marotta, Mattia Marte, Elena Mazzalai, Irma Germani, Arianna Bellini, Andrea Bongiovanni, Marta Castellani, Ludovica D’Agostino, Andrea De Giorgi, Chiara De Marchi, Elisa Mazzeo, Shadi Orlandi, Matteo Piattoli, Eleonora Ricci, Leonardo Maria Siena, Alessandro Territo, Stefano Zanni

**Affiliations:** 1 Department of Public Health and Infectious Diseases, Sapienza University of Rome, 00185 Rome, Italy; claudia.isonne@uniroma1.it (C.I.); giuseppe.migliara@uniroma1.it (G.M.); mariateresa.ceparano@uniroma1.it (M.C.); giancarlo.ceccarelli@uniroma1.it (G.C.); maria.degiusti@uniroma1.it (M.D.G.); corrado.devito@uniroma1.it (C.D.V.); carolina.marzuillo@uniroma1.it (C.M.); paolo.villari@uniroma1.it (P.V.); laviniacamilla.barone@uniroma1.it (L.C.B.); dara.giannini@uniroma1.it (D.G.); daniela.marotta@uniroma1.it (D.M.); mattia.marte@uniroma1.it (M.M.); elena.mazzalai@uniroma1.it (E.M.); arianna.bellini@uniroma1.it (A.B.); andrea.bongiovanni@uniroma1.it (A.B.); marta.castellani@uniroma1.it (M.C.); ludovica.dagostino@uniroma1.it (L.D.); andrea.degiorgi@uniroma1.it (A.D.G.); chiara.demarchi@uniroma1.it (C.D.M.); elisa.mazzeo@uniroma1.it (E.M.); shadi.orlandi@uniroma1.it (S.O.); eleonora.ricci@uniroma1.it (E.R.); leonardo.siena@uniroma1.it (L.M.S.); alessandro.territo@uniroma1.it (A.T.); stefano.zanni@uniroma1.it (S.Z.); 2 Department of Anaesthesia and Intensive Care Medicine, Umberto I Teaching Hospital, Sapienza University of Rome, 00161 Rome, Italy; francesco.alessandri@uniroma1.it (F.A.); guglielmo.tellan@uniroma1.it (G.T.); f.pugliese@uniroma1.it (F.P.); irmager@libero.it (I.G.); matteo.piattoli@uniroma1.it (M.P.); 3 Department of General and Specialist Surgery “P. Stefanini”, Sapienza University of Rome, 00185 Rome, Italy

**Keywords:** healthcare-associated infection, devices-related infection, COVID-19, intensive care unit, COVID-19 pandemic, SARS-CoV-2

## Abstract

The COVID-19 pandemic has increased the healthcare-associated infection (HAI) risk in intensive care unit (ICU) patients. However, a comparison between patients with and without COVID-19 in terms of HAI incidence has been rarely explored. In this study, we characterized the occurrence of HAI among patients with and without COVID-19 admitted to the ICU of the Umberto I hospital of Rome during the first 16 months of the pandemic and also identified risk factors for HAI acquisition. Patients were divided into four groups according to their ICU admission date. A multivariable conditional risk set regression model for multiple events was constructed for each admission period. Adjusted hazard ratios and 95% confidence intervals were calculated. Overall, 352 COVID-19 and 130 non-COVID-19 patients were included, and a total of 361 HAIs were recorded. We found small differences between patients with and without COVID-19 in the occurrence and type of HAI, but the infections in the two cohorts mostly involved different microorganisms. The results indicate that patient management was likely an important factor influencing the HAI occurrence during the pandemic. Effective prevention and control strategies to reduce HAI rates should be implemented.

## 1. Introduction

The coronavirus disease 2019 (COVID-19) pandemic has imposed many challenges on healthcare systems worldwide [1]. On the one hand, healthcare facilities have had to face an increasing number of COVID-19 patients, who often required hospital admission [2]; on the other hand, they had to ensure healthcare delivery for non-COVID-19 patients, who continued to need care [3,4,5]. Healthcare activities were rapidly reorganized, and some wards, especially intensive care units (ICUs), had to increase their capacity [6,7]. Within this context, the application of appropriate measures to prevent and control healthcare-associated infections (HAIs) has been particularly critical, with both surveillance efforts and containment strategies sometimes failing [8,9].

Recent evidence shows that patients hospitalized in ICUs during the pandemic have been at increased risk of HAIs [10,11]. Infection control practices may have been hindered by a fear among healthcare workers of being infected and severe staffing shortage, with a consequent reallocation to ICU wards of personnel not previously trained in critical care [12,13,14]. Additionally, the clinical characteristics of COVID-19 patients, often requiring the use of invasive devices and a long ICU stay, could have contributed to the increase in HAIs [15,16]. Furthermore, the frequent use of antibiotics, especially in COVID-19 patients, might have accentuated antimicrobial resistance and the incidence of multidrug-resistant organisms in ICUs [17]. However, non-COVID-19 patients could also have been at high risk of HAI given their health conditions [18]. In fact, the rationalization of ICU beds because of the pandemic may have determined a change in the pattern of patient admission, limiting it to extremely critical patients who could not be managed in other wards [19,20,21].

A growing number of studies have investigated the incidence of HAIs among COVID-19 patients, addressing factors and outcomes relating to their occurrence [6,10,22,23,24,25]. The data have highlighted some changes in HAI type and the pathogens involved with the advent of the pandemic [22,25,26,27], but a direct comparison of patients hospitalized with and without COVID-19 during the pandemic is still lacking [28]. The aim of the study was twofold: (i) to characterize over time the occurrence of HAIs in COVID-19 and non-COVID-19 patients admitted to the main ICU of Umberto I teaching hospital of Rome during the first 16 months of the pandemic and (ii) to identify key factors associated with onset of such HAIs. The findings should help to assess the impact of the pandemic on HAIs and lead to the design and implementation of targeted prevention strategies.

## 2. Materials and Methods

### 2.1. Patients and Data Collection

In this study, we retrospectively analyzed COVID-19 and non-COVID-19 patients admitted to the main ICU of Umberto I teaching hospital of Rome from 1 March 2020 to 6 June 2021. The follow-up of both cohorts terminated on 30 June 2021. Data about HAIs were collected prospectively and retrieved from the active HAI surveillance system that has been conducted since May 2016 by the Department of Public Health and Infectious Diseases [29]. The surveillance system is based on a protocol derived from the National Healthcare Safety Network protocol of the Center for Disease Control [30] and the European Center for Disease Prevention and Control [31]. All patients hospitalized in the ICU for at least 48 h are monitored until their discharge from the ICU. The incidence of blood infections involving central lines (catheter-related bloodstream infections, CRBSIs), pneumonia associated with mechanical ventilation (ventilation-associated pneumonia, VAP), and urinary tract infections associated with bladder catheters (catheter-associated urinary tract infections, CAUTIs) that occurred more than 48 h after device insertion was registered. The surveillance system also routinely stores data on the incidence of bloodstream infections of unknown origin (BUO) and surgical site infections (SSIs) that occur 48 h after ICU admission or within 30 days after surgery, respectively. 

A form with four parts was used to systematically collect data. The first section refers to patient demographics and information on hospitalization (date of ICU admission, type of ICU admission, discharge date, status of the patient at discharge, pre-existing comorbidities, Simplified Acute Physiology Score (SAPS) II). The second section regards exposure to risk factors: time of the patient’s exposure to urinary catheterization, central venous catheterization, and mechanical ventilation. It is also specified whether the device was present within the 48 h prior to the onset of infection. The third section focuses on antibiotic therapy (antibiotic class and duration and route of administration). The last section investigates the diagnosed HAIs and microbiological cultures performed: site of infection, date of HAI onset and microbiological confirmation (date of sample collection and microorganisms isolated). A positive result of real-time reverse transcriptase-polymerase chain reaction assay of nasal and pharyngeal swabs was considered for laboratory confirmation of SARS-CoV-2 in COVID-19 patients. Antibiotic consumption was coded as having used any antibiotic agent for at least two days via systemic administration in the period from ICU admission to the day before HAI onset or the date of discharge.

The institutional ethics board of the Umberto I teaching hospital of Rome approved this study (protocol n. 800/2020).

### 2.2. Statistical Analysis

Patients were divided into four groups according to their ICU admission date (period I: from 1 March 2020 to 10 May 2020; period II: from 11 May 2020 to 23 August 2020; period III: from 24 August 2020 to 2 February 2021; and period IV: from 3 February 2021 to 6 June 2021). Descriptive statistics were obtained using means and standard deviations for continuous variables and proportions for dichotomous and categorical variables. The ICU mortality rate and the associated Poisson 95% confidence interval (CI) was calculated per 1000 patient-days. Time-to-HAI was estimated through survival analysis. Given the occurrence of multiple HAIs in some patients, we used a multivariable conditional risk set regression model for multiple events (Prentice, Williams, and Peterson Total Time (PWP-TT) model) to explore the effect of the exposure of interest on the outcome [32], providing estimates of adjusted hazard ratio (aHR) and its associated 95% CI. When more than one HAI was diagnosed simultaneously in the same patient, they were considered as a single event. A total of three models were built to regress the HR of HAIs (i.e., one for each period, except for period II, which was excluded since no HAI occurred among COVID-19 patients). The main exposure of interest (i.e., being or not being a COVID-19 patient) was adjusted for the same covariates in all models by including the potential confounders of the association [31]. Only the most frequent comorbidities (i.e., hypertension and diabetes mellitus) and antibiotic exposures (i.e., carbapenems, extended spectrum cephalosporins, glycopeptides, macrolides, penicillins, and polymyxins) were considered. Since days of central venous catheterization, days of urinary catheterization, and days of mechanical ventilation were collinear (variance inflation factor >5), only the latter was kept for further analyses. Missing values for SAPS II (29.1%) were imputed by univariate multiple imputation using a truncated linear regression model constraining the imputation between 0 and 163, the lowest and highest possible scores for SAPS II. In addition to the outcome, all the variables used in the multivariable survival analysis were included in the imputation models. To account for the high rate of missing values, 50 imputed datasets were generated [33].

As a result, the final regression models included the following variables: COVID-19 (no/yes), age (years, continuous), gender (female/male), SAPS II (continuous), hypertension (no/yes), diabetes mellitus (no/yes), previous exposure to carbapenems (no/yes), extended spectrum cephalosporins (no/yes), previous exposure to glycopeptides (no/yes), previous exposure to macrolides (no/yes), previous exposure to penicillins (no/yes), previous exposure to polymyxins (no/yes), and mechanical ventilation (days, continuous). The proportionality assumption was checked by testing the statistical significance of interaction terms involving failure time. 

All analyses were performed using STATA (StataCorp LLC, 4905 Lakeway Drive, College Station, TX, USA), version 17.0. A two-sided *p*-value < 0.05 was considered statistically significant.

## 3. Results

### 3.1. Characteristics of the Patients

A total of 352 COVID-19 and 130 non-COVID-19 patients were analyzed (Table 1). Overall, the COVID-19 cohort was more represented throughout the study periods except for period II, where the vast majority were non-COVID-19 patients (period I: 47 vs. 18 patients; period II: 4 vs. 45 patients; period III: 130 vs. 33 patients; and period IV: 171 vs. 34 patients, respectively). Similarly, the cumulative observation time from ICU admission to the end of follow-up was longer in the COVID-19 cohort in all but period II (period I: 800 vs. 301 days; period II: 61 vs. 932 days; period III: 2179 vs. 894 days; and period IV: 2734 vs. 720 days, respectively). In both cohorts, women were less frequently hospitalized throughout the study period apart from period II, when gender was equally distributed in both groups (50.0% and 48.9%, respectively), and period IV for the non-COVID-19 group (50%). Patients with COVID-19 seemed to be older than non-COVID-19 patients in the first two periods only (69 vs. 65 years, and 72.3 vs. 61.5 years, respectively). In period I, patients admitted to the ICU came mostly from hospital wards (46.8% of COVID-19 patients and 67.0% of non-COVID-19 patients), whereas in the other time periods, the largest proportion of patients were admitted mainly from the emergency department (from 47.1% in period IV to 75.0% in period II). The mean SAPS II score was lower in COVID-19 than in non-COVID-19 patients, ranging between 33.3 and 37.3 in the COVID-19 group and between 36.6 and 50.0 in the non-COVID-19 group. Comorbidities were reported heterogeneously: apart from COVID-19 patients in period II, hypertension was the most frequently found comorbidity in both cohorts, ranging from 30.3% to 51.1% and from 26.7% to 41.2% in the COVID-19 and non-COVID-19 cohorts, respectively, followed by diabetes mellitus, obesity, chronic obstructive pulmonary disease, and active cancer. 

Deaths occurred more frequently in COVID-19 patients, for whom a higher ICU mortality rate was found (range: 0.03 per 1000 patient-days (95% CI: 0.02–0.03) to 0.05 per 1000 patient-days (95% CI: 0.02–0.15). Additionally, COVID-19 patients had a shorter ICU stay compared to the non-COVID-19 cohort (range: 15.3–17.0 days vs. 16.7–27.1 days, respectively) and a shorter mean use of invasive devices (i.e., central venous catheter, urinary catheter, and mechanical ventilation) in all time periods except for period I, where they were both slightly higher in this subgroup (length of ICU stay: 17.0 vs. 16.7 days; range of device mean use: 12.8 to 17.0 days vs. 10.9 to 16.7 days, respectively). Invasive ventilation was mostly required in COVID-19 cases in periods I and III, whereas in the non-COVID-19 cohort, it was most frequently required in periods II and IV.

As for antibiotic consumption, there was a high consumption of glycopeptides and penicillins plus beta lactamase inhibitors in the COVID-19 cohort, whereas the other antibiotic classes (carbapenems, extended spectrum cephalosporins, macrolides, and polymixins) were heterogeneously prescribed throughout the study period in both cohorts.

### 3.2. Occurrence and Characteristics of HAIs

**Figure 1:**
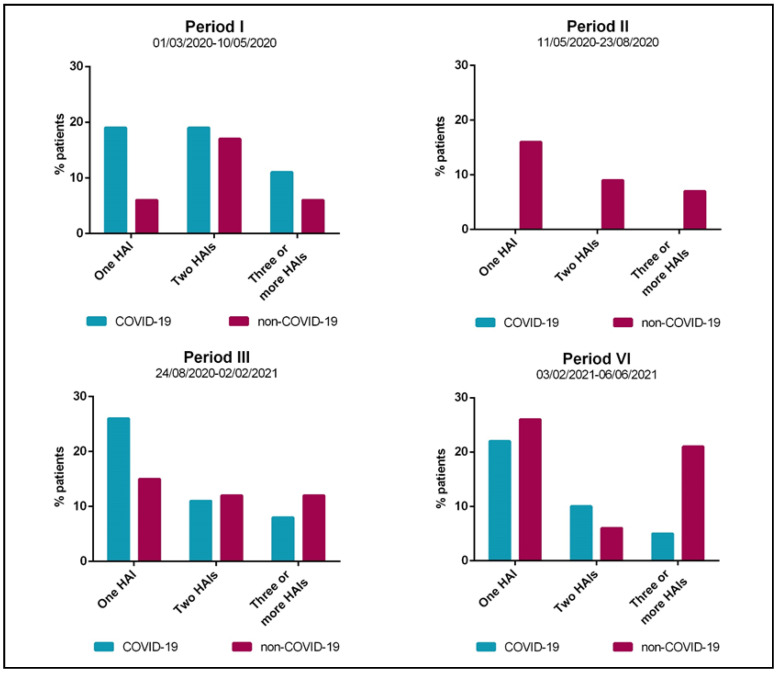
Cumulative incidence of patients with at least one healthcare-associated infection (HAI) admitted to the Intensive Care Unit of Umberto I teaching hospital of Rome between 1 March 2020 and 6 June 2021 by study period.

A few differences between the two cohorts in the cumulative incidence of patients with at least one HAI were observed during the four periods (Figure 1). During the first months of the emergency, compared to the non-COVID-19 group, higher proportions of COVID-19 patients developed one, two, and three or more HAIs (19.1% vs. 6.0%; 19.1% vs. 17.0%; and 11.0% vs. 6.0%, respectively), while none of them developed a HAI during the second period. In addition, whereas a higher proportion of COVID-19 patients developed one HAI during the third period (26.2% vs. 15.2%), they seemed to be less affected by multiple infections. Lastly, during the fourth period, the non-COVID-19 group were more likely to develop one HAI (27.0 vs. 22.2%) or three or more HAIs (21.0% vs. 5.3%).

**Figure 2:**
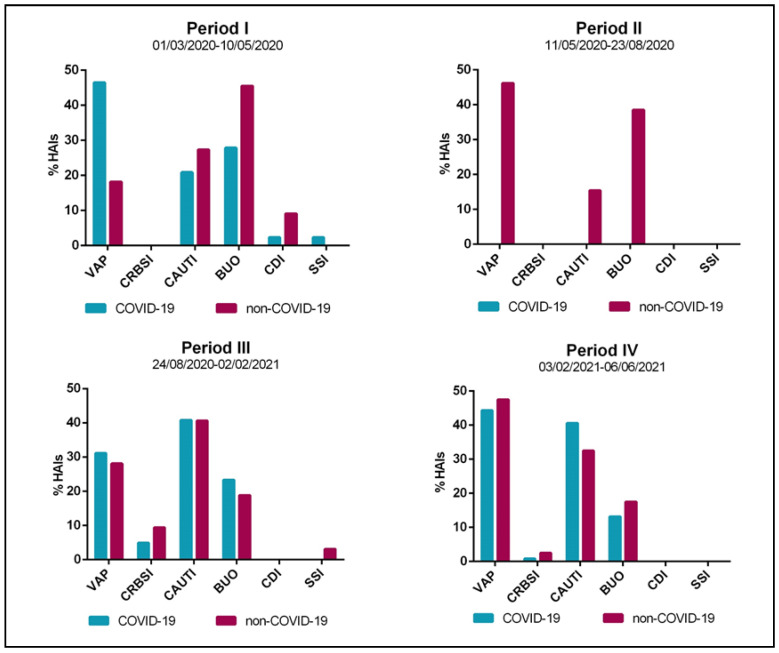
Characteristics of healthcare-associated infections (HAIs) diagnosed in patients admitted to the Intensive Care Unit of Umberto I teaching hospital of Rome between 1 March 2020 and 6 June 2021 by study period. VAP, ventilation-associated pneumonia; CRBSI, catheter-related bloodstream infection; CAUTI, catheter-associated urinary tract infection; BUO, bloodstream infections of unknown origin; CDI, *Clostridium difficile* infection; SSI, surgical site infection.

A total of 54 HAIs were recorded in period I, 26 in period II, 135 in period III, and 146 in period IV. They occurred mainly in COVID-19 patients in periods I, III, and IV (*N* = 43, *N* = 103 and *N* = 106, respectively), whereas non-COVID-19 patients were only affected during period II. As for the HAI type, during period I, the COVID-19 cohort had a high incidence of VAP (46.6%), whereas a high number of BUO were diagnosed among non-COVID-19 patients (45.5%) (Figure 2). In period II, most infections were VAP and BUO (46.2% and 38.5%, respectively). In the last two periods, the type of HAI was similar in both cohorts, with a high incidence of CAUTI in period III (40.8% vs. 40.6%) and VAP in period IV (44.3% vs. 47.5%). Lastly, infections sustained by *Clostridium difficile* and SSIs were rarely diagnosed and almost only in period I in both cohorts.

**Figure 3:**
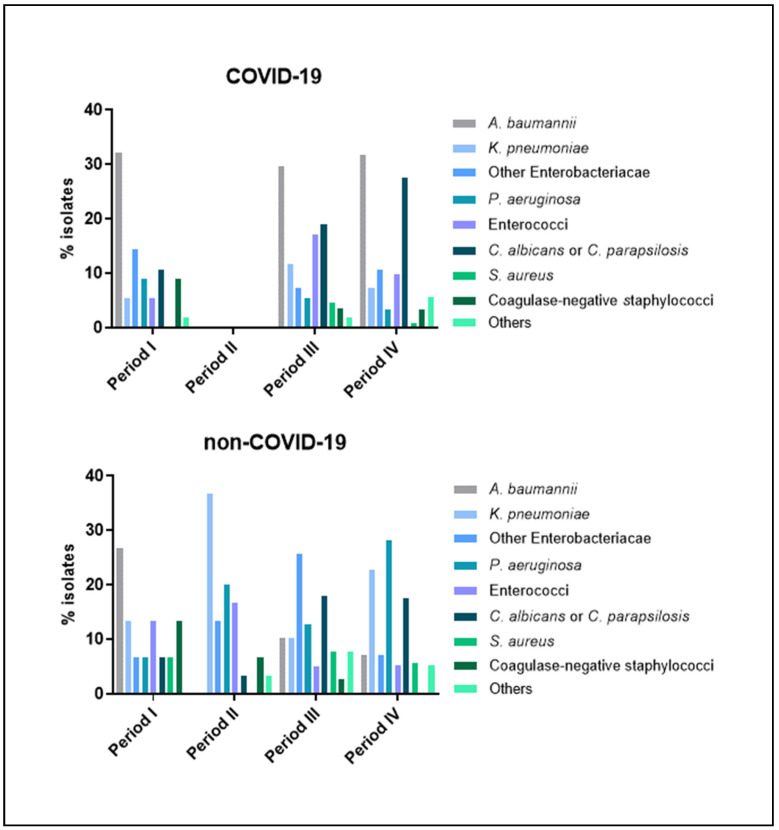
Microorganisms responsible for healthcare-associated infections (HAIs) isolated in patients admitted to the Intensive Care Unit of Umberto I teaching hospital of Rome between 1 March 2020 and 6 June 2021.

Among COVID-19 patients, HAIs were mostly caused by *Acinetobacter baumannii* in all periods (around 30% each), followed by other *Enterobacteriaceae* in period I and *Candida albicans* or *C. parapsilosis* in periods III and IV, respectively (Figure 3). By contrast, among non-COVID-19 patients, *A. baumannii* was primarily responsible for HAIs during period I only (26.7%), whereas *Klebsiella pneumoniae* and *Pseudomonas aeruginosa* were the most frequently isolated pathogens during period II (26.7% and 20.0%, respectively) and period IV (22.8% and 28.1%, respectively) and other *Enterobacteriaceae* and *C. albicans* or *C. parapsilosis* in period III (25.6% and 18.0%, respectively). *Staphylococcus aureus*, coagulase-negative staphylococci, and the other microorganisms were less frequently detected in both cohorts.

### 3.3. Risk Factors for HAI

In multivariable analyses, COVID-19 was found to be positively associated with HAI in patients admitted to the ICU during period III only (aHR: 2.43, 95% CI: 1.26–4.67), when being older also seemed to be a risk factor (aHR: 1.03, 95% CI: 1.01–1.04). Sex, SAPS II score, hypertension, and diabetes mellitus did not seem to be independent predictors of HAIs in any time period. By contrast, higher exposure to mechanical ventilation was associated with a reduction in the risk of HAI in periods I and IV only (aHR: 0.86, 95% CI: 0.81–0.92 and aHR: 0.94, 95% CI: 0.92–0.96, respectively). Among antibiotics, a lower risk of HAI was found for previous consumption of carbapenemes and penicillins plus beta-lactamase inhibitors in periods III and IV (period I: aHR: 0.54, 95% CI: 0.35–0.84 and aHR: 0.50, 95% CI: 0.31–0.81; period III: aHR: 0.60, 95% CI: 0.39–0.93 and aHR: 0.53, 95% CI: 0.34–0.84, respectively). Conversely, glycopeptides during period III and macrolides during period IV seemed to negatively influence the occurrence of HAI (aHR: 0.20, 95% CI: 0.08–0.49 and aHR: 0.56, 95% CI: 0.35–0.91, respectively) even though the protective effect of glycopeptide administration seemed to reduce over time. Lastly, extended spectrum cephalosporins and polymixins had no influence on HAIs in any period (Table 2).

## 4. Discussion

Evidence from the literature suggests that COVID-19 patients have an increased risk of HAIs for a number of reasons, including their clinical condition, which often requires the use of invasive devices; a long ICU stay; and high rates of antibiotic administration, but also because of difficulties in their management as a result of the reorganization of healthcare facilities [9,11,22,34]. In line with these findings, we have already described an overall increase in the incidence of patients with HAIs in our ICU in a previous study [22] in which we compared the patients admitted between March and April 2020 to patients hospitalized one year before. However, our multivariable analysis did not confirm a higher susceptibility of COVID-19 patients to HAIs compared to the non-COVID-19 cohort admitted in the same period, probably because of reduced statistical power [22]. Therefore, in this study, we decided to expand the enrollment period to include the patients with and without COVID-19 admitted during the first 16 months of the pandemic, and we were able to confirm a higher susceptibility of the COVID-19 cohort to HAIs but only during some of the periods analyzed. As for the first period, similarly to our previous results [22], we observed a slightly higher incidence of HAIs in these patients that was not confirmed by the multivariable analysis probably because of the low sample size. We have already argued that in those early months of the pandemic, the lack of knowledge about the SARS-CoV-2 virus, the fear of becoming infected, and the shortage of personal protective equipment could have reduced the compliance of healthcare workers with hygiene precautions, increasing the risk of cross-contamination and thus facilitating the growth of microorganisms, especially among COVID-19 patients [35,36]. Additionally, healthcare systems faced severe staffing shortages likely due to the healthcare professionals’ exposure to the virus, illness, or the need to care for family members at home with consequent non-adequate staffing to patient ratios, another factor that could have contributed to HAI onset [37]. On the other hand, the absence of HAIs in our COVID-19 cohort during the summer of 2020 could be the result of the national lockdown in Italy over the previous months [38], which significantly reduced virus spread and consequently hospital admissions of infected patients [39]. Conversely, compared to the other cohort, the COVID-19 patients seemed to be at higher risk of HAIs in autumn–winter 2020, when the number of SARS-CoV-2 infections peaked, and the consequent hospitalizations dramatically impacted their ICU management due to staff work overload and reduced availability of hospital beds, making particularly critical the application of HAI prevention and control strategies [40]. Nevertheless, during the fourth period, multivariable analysis revealed a similar occurrence of HAIs in the two subgroups, suggesting that the psychological effect of the vaccination campaign and a better organization of the ICU ward, which had already faced the previous wave of the pandemic, may have limited cross-contamination among COVID-19 patients [41]. Hence, since these findings suggest that healthcare personnel took a different approach to managing these patients during the pandemic and that this may have had a significant role in preventing HAI acquisition, a proper reorganization and restructuring of the ICU should be planned to ensure adequate healthcare delivery in emergency situations [5]. 

Among other factors that may influence the onset of HAIs, variables relating to the demographic characteristics of patients (e.g., age, sex) or their clinical conditions (e.g., SAPS II, comorbidities) were found to increase the risk albeit not consistently [42,43]. Data on this issue during the pandemic are still limited [28], but, in line with the available literature [44,45], none of these factors seemed to play a major role in acquisition of HAIs in our study. The exception was mechanical ventilation, which seemed to be protective, probably because of the depletion of susceptible patients, a selection bias typical of survival analysis. Furthermore, in contrast to other reports [46,47], we found that antibiotic consumption had a negative impact on HAIs acquisition although heterogeneously between classes of antibiotics and across periods. Interestingly, we found glycopeptides and macrolides among the protective factors; these were mostly prescribed in COVID-19 patients because of their therapeutic effect against SARS-CoV-2 pneumonia [48,49]. However, given the extensive consumption of multiple antibiotic classes in all patients, these results should be interpreted with caution. Indeed, antibiotic use is universally recognized as a major cause of antimicrobial resistance [50]. Therefore, widespread action is needed to implement antimicrobial stewardship programs that optimize and target antibiotic consumption in healthcare settings to reduce the emergence and spread of multidrug-resistant bacteria [50]. 

As for HAI type, most were device related in both groups. This is not unexpected, given the frequently severe conditions of our patients and the high rates of invasive device use that were recorded. Particularly, the COVID-19 cohort seemed to be critically compromised, as indicated by the higher mortality rates and the shorter length of stay and use of devices observed. However, we did not observe any substantial difference in HAI breakdown between the two cohorts except for the first months of the emergency, in which VAP affected mainly COVID-19 patients. Current literature has already argued that the pathophysiology of SARS-CoV-2 pneumonia [23] together with the challenges relating to its diagnosis [51] and the treatment used for these patients (i.e., the frequent need for mechanical ventilation) [52] could promote VAP onset as we previously described [22]. Indeed, despite the application of a less invasive therapeutic approach in the following months [53,54] and the early administration of new targeted therapies [55], COVID-19 patients remained at high risk of VAP, probably because of the effect of SARS-CoV-2 virus on immunity [56]. By contrast, we found that different microorganisms were circulating in the two cohorts. The reason for such a difference could be cross-contamination in the rooms in which the two groups were hospitalized; i.e., there might have been indirect transmission of microorganisms via the contaminated environment and healthcare personnel [57]. In this regard, it is important to mention that the two cohorts were physically separated, each with its own dedicated ICU staff. Additionally, *A. baumannii, K. pneumoniae,* and *P. aeruginosa,* mostly detected within COVID-19 and non-COVID-19 patients, are known to persist for long periods on hospital surfaces [58,59,60]. Hence, given that these organisms are often multidrug resistant [61] and that associated patient infections result in increased length of stay, costs, and mortality [62], improvements in environmental cleaning and disinfection in hospital settings are essential. However, healthcare professionals must also increase their awareness of and adherence to hygiene precautions to limit the spread of microorganisms as much as possible [57,61,63,64].

This study has several strengths and limitations. The main strength is the ability to compare data over time: since the data collected represent part of an ongoing four-year surveillance system routinely carried out by the Department of Public Health and Infectious Diseases, a potential distortion of the results due to work overload of ICU staff is unlikely. Furthermore, to the best of our knowledge, this is the first study that followed COVID-19 and non-COVID-19 patients over a long period, comparing the occurrence of HAIs and investigating HAI type and the microorganisms responsible for their onset in the two cohorts. In addition, since we used the PWP-TT model, we were able to incorporate information relating to all HAIs that occurred throughout the study period. By contrast, the first limitation is the low number of patients, especially in the non-COVID-19 group, which may have limited the statistical power. Secondly, patients discharged from the ICU were no longer under surveillance although only the most stable patients were chosen for transfer. Thirdly, to adjust for patient clinical severity, we used multiple imputation to generate missing values for SAPS II, compensating for the high rate of missing values by imputing 50 datasets. Lastly, we did not investigate the HAI impact on patient mortality although this was not a goal of our research. Further studies should be conducted to address this issue.

## 5. Conclusions

Small differences between patients with and without COVID-19 were found in HAI occurrence and type for a few periods only, whereas different microorganisms were circulating within the two cohorts throughout the study. These results suggest a crucial role for patient management and highlight the importance of implementing effective HAI prevention and control strategies. To improve healthcare delivery, further efforts are needed to promote adherence to hygiene precautions and to increase knowledge and awareness of these issues among healthcare workers.

## Figures and Tables

**Table 1:** Characteristics of the patients admitted to the Intensive Care Unit (ICU) of Umberto I teaching hospital of Rome between 1 March 2020 and 6 June 2021 by study period. Results are expressed as number (percentage) or mean (standard deviation).

	Period I		Period II		Period III		Period IV	
	1 March 2020 to 10 May 2020		11 May 2020 to 23 August 2020		24 August 2020 to 2 February 2021		3 February 2021 to 6 June 2021	
	With	Without	With	Without	With	Without	With	Without
	COVID-19	COVID-19	COVID-19	COVID-19	COVID-19	COVID-19	COVID-19	COVID-19
Patients	47	18	4	45	130	33	171	34
Observation time, person-days	800	301	61	932	2179	894	2734	720
Gender (female)	16 (34.0)	7 (38.9)	2 (50.0)	22 (48.9)	41 (31.5)	12 (36.4)	59 (34.5)	17 (50.0)
Age, years	69 (13.0)	65 (16.0)	72.3 (17.0)	61.5 (16.2)	61.2 (13.1)	64.3 (18.2)	59.2 (13.9)	68.2 (13.9)
Admission to the ICU								
Ward	22 (46.9)	12 (66.7)	1 (25.0)	12 (26.7)	42 (33.0)	13 (39.4)	36 (21.1)	17 (50.0)
Other hospital	4 (8.5)	0 (0.0)	0 (0.0)	2 (4.4)	0 (0.0)	1 (3.0)	26 (15.3)	1 (2.9)
Emergency Department	21 (44.7)	6 (33.3)	3 (75.0)	31 (68.9)	68 (52.3)	15 (45.5)	109 (63.8)	16 (47.1)
SAPS II Score (*N* = 341)	37.3 (9.6)	36.6 (20.8)	33.3 (11.1)	38.7 (15.9)	34.6 (11.4)	45.4 (13.7)	35.8 (10.7)	50 (14.0)
Coexisting conditions								
Hypertension	24 (51.1)	5 (27.8)	0 (0.0)	12 (26.7)	54 (41.5)	10 (30.3)	66 (38.6)	14 (41.2)
Diabetes mellitus	7 (14.9)	2 (11.1)	0 (0.0)	5 (11.1)	26 (20.0)	3 (9.1)	29 (17.0)	6 (17.6)
Obesity (BMI ≥ 30)	3 (6.4)	1 (5.6)	0 (0.0)	2 (4.4)	18 (13.9)	2 (6.1)	31 (18.1)	5 (14.7)
COPD	2 (4.3)	3 (16.7)	0 (0.0)	5 (11.1)	16 (12.3)	4 (12.1)	5 (2.9)	2 (5.9)
Asthma	3 (6.4)	1 (5.6)	0 (0.0)	1 (2.2)	4 (3.1)	0 (0.0)	3 (1.8)	0 (0.0)
Coronary heart disease	5 (10.6)	2 (11.1)	0 (0.0)	1 (2.2)	12 (9.2)	1 (3.0)	13 (7.6)	2 (5.9)
Chronic kidney disease	2 (4.3)	1 (5.6)	0 (0.0)	2 (4.4)	10 (7.7)	1 (3.0)	5 (2.9)	2 (5.9)
Chronic liver disease	0 (0.0)	0 (0.0)	0 (0.0)	4 (8.9)	1 (0.8)	0 (0.0)	0 (0.0)	1 (2.9)
Active cancer	6 (12.8)	0 (0.0)	0 (0.0)	2 (4.4)	14 (10.8)	4 (12.1)	15 (8.8)	4 (11.8)
Immunodeficiency	1 (2.1)	0 (0.0)	0 (0.0)	0 (0.0)	3 (2.3)	0 (0.0)	0 (0.0)	0 (0.0)
ICU deaths	30 (63.8)	6 (33.3)	3 (75.0)	10 (22.0)	80 (61.5)	9 (27.3)	74 (43.3)	9 (26.5)
Mortality rate (95% CI) per 1000 patient-days	0.04 (0.03–0.05)	0.02 (0.01–0.04)	0.05 (0.02–0.15)	0.01 (0.01–0.02)	0.04 (0.02–0.04)	0.01 (0.01–0.02)	0.03 (0.02–0.03)	0.01 (0.01–0.02)
Length of ICU stay, days	17.0 (13.5)	16.7 (25.6)	15.3 (10.4)	20.7 (15.2)	16.8 (11.9)	27.1 (39.0)	16.0 (14.7)	21.2 (16.5)
Central venous catheter, days	15.4 (14.4)	14.6 (26.3)	11.5 (11.8)	19.0 (16.4)	10.0 (11.4)	17.2 (22.2)	11.3 (13.2)	19.7 (15.7)
Urinary catheter, days	16.0 (14.2)	15.2 (26.2)	15.3 (10.4)	20.0 (15.0)	16.2 (12.0)	20.0 (23.5)	15.7 (13.6)	19.7 (16.7)
Invasive ventilation, days	12.8 (8.9)	10.9 (12.5)	19.5 (13.4)	23.7 (29.8)	18.4 (35.4)	22.0 (26.1)	16.1 (14.7)	18.1 (16.3)
Patients with invasive ventilation	40 (85.1)	13 (72.2)	2 (50.0)	40 (88.9)	109 (83.8)	26 (78.8)	99 (57.9)	32 (94.1)

CI, confidence interval; SAPS II, Simplified Acute Physiology Score II; BMI, body mass index; COPD, chronic obstructive pulmonary disease.

**Table 2:** Multivariable conditional risk set regression models for time to healthcare-associated infection (HAI) occurred among the patients admitted to the Intensive Care Unit of Umberto I teaching hospital of Rome between 1 March 2020 and 6 June 2021 by study period.

	Period I		Period III		Period IV	
	1 March 2020–10 May 2020		24 August 2020–2 February 2021		3 February 2021–6 June 2021	
	aHR (95% CI)	*p*-Value	aHR (95% CI)	*p*-Value	aHR (95% CI)	*p*-Value
COVID-19	1.19 (0.25–5.67)	0.823	2.43 (1.26–4.67)	0.008	0.84 (0.48–1.46)	0.531
Age (years)	0.99 (0.94–1.03)	0.553	1.03 (1.01–1.04)	0.001	1.01 (0.99–1.03)	0.317
Sex (male)	2.50 (0.88–7.10)	0.085	0.93 (0.65–1.35)	0.717	1.16 (0.78–1.71)	0.460
SAPS II	0.98 (0.93–1.03)	0.410	1.00 (0.98–1.03)	0.772	1.01 (0.99–1.04)	0.334
Hypertension	0.82 (0.30–2.22)	0.696	0.93 (0.62–1.39)	0.719	1.01 (0.66–1.54)	0.967
Diabetes mellitus	0.87 (0.22–3.50)	0.845	0.32 (0.10–1.06)	0.061	1.29 (0.82–2.03)	0.272
Invasive ventilation, days	0.86 (0.81–0.92)	<0.001	1.00 (1.00–1.01)	0.546	0.94 (0.92–0.96)	<0.001
Carbapenems	0.42 (0.17–1.08)	0.073	0.54 (0.35–0.84)	0.006	0.60 (0.39–0.93)	0.024
Extended-spectrum cephalosporins	0.40 (0.12–1.32)	0.133	0.63 (0.34–1.16)	0.136	0.89 (0.58–1.37)	0.598
Glycopeptides	0.36 (0.12–1.03)	0.057	0.20 (0.08–0.49)	<0.001	0.67 (0.43–1.04)	0.077
Penicillins	1.13 (0.24–5.38)	0.880	0.50 (0.31–0.81)	0.005	0.53 (0.34–0.84)	0.007
Polymixins	0.79 (0.35–1.80)	0.576	0.64 (0.38–1.08)	0.097	0.68 (0.44–1.06)	0.088
Macrolides	0.65 (0.26–1.63)	0.356	0.80 (0.49–1.31)	0.381	0.56 (0.35–0.91)	0.018
Age * time	1.00 (1.00–1.01)	0.030				
Glycopeptides * time			1.08 (1.03–1.12)	<0.001		
Diabetes mellitus * time			1.13 (1.04–1.22)	0.002		

aHR, adjusted hazard ratio; CI, confidence interval; SAPS II, Simplified Acute Physiology Score II; * interaction term.

## Data Availability

Data available on reasonable request due to privacy reasons.
